# Comparison of the efficiency, safety, and survival outcomes in two stem cell mobilization regimens with cyclophosphamide plus G-CSF or G-CSF alone in multiple myeloma: a meta-analysis

**DOI:** 10.1007/s00277-020-04376-w

**Published:** 2021-01-06

**Authors:** Liwen Wang, Hongxian Xiang, Yuhan Yan, Zuqun Deng, Hui Li, Xin Li, Jing Liu

**Affiliations:** 1 Department of Hematology, Third Xiangya Hospital, Central South University, Changsha, 410013 Hunan Province China; 2 Xiangya School of Medicine, Central South University, Changsha, 410013 Hunan Province China; 3 Assisted Reproduction Unit, Department of Obstetrics and Gynecology, Sir Run Shaw Hospital, School of Medicine, Zhejiang University, Hangzhou, 310016 Zhejiang Province China

**Keywords:** Multiple myeloma, Cyclophosphamide, Granulocyte-colony stimulating factor, Stem cell mobilization, Event-free survival

## Abstract

Autologous stem cell transplantation as a frontline treatment for patients with multiple myeloma (MM) requires an adequate peripheral blood stem cell (PBSC) collection before processing. Granulocyte-colony stimulating factor (G-CSF) with or without cyclophosphamide (CTX) is a common regimen for PBSC mobilization; their benefits and risks are controversial. To compare the efficiency, safety, and survival outcomes between the two regimens, we conducted a meta-analysis including 18 studies with 4 prospective and 14 retrospective studies; a total of 2770 patients with MM were analyzed. The CTX plus G-CSF regimen had higher yields of total CD34^+^ cells (SMD = 0.39, 95% CI (0.30, 0.49)), and higher mobilization rates of the target ⩾ 2 × 10^6^/kg (OR = 3.34, 95% CI (1.82, 6.11)) and 4 × 10^6^/kg (OR = 2.16, 95% CI (1.69, 2.76)) cells. A favorable event-free survival (EFS) (HR = 0.73, 95% CI (0.58, 0.93), *p* = 0.01) and better 3-year EFS rate (OR = 1.65, 95% CI (1.1, 2.47), *p* = 0.02) were also reached in the patients with CTX plus G-CSF mobilization, although the risks of admission (OR = 26.49, 95% CI (7.31, 95.97)) and fever (OR = 13.66, 95% CI (6.21, 30.03)) during mobilization were increased, the treatment-related mortality was consistent (*p* = 0.26). The CTX plus G-CSF regimen was superior to the G-CSF-alone regimen for PBSC mobilization in patients with MM.

**Supplementary Information:**

The online version contains supplementary material available at 10.1007/s00277-020-04376-w.

## Background

The estimated incidence of multiple myeloma (MM) is currently 160,000, and mortality amounts to 106,000 worldwide [1]. In the USA, MM is the second common hematological malignancy, which accounts for 2.1% of all cancer-related death [2]. Survival estimates in MM are varied due to different source of the data; some randomized controlled trials (RCTs) demonstrated that the median overall survival (OS) in MM is approximately 6 years [3], and for patients with autologous stem cell transplantation (ASCT) eligible is around 8 years [4]. With further advances in the MM treatment landscape, including the development and introduction of potential new drugs, like proteasome inhibitors (PIs), immunomodulatory agents (IMiDs), antibody agents, and chimeric antigen receptor T (CART) therapy, survival in MM has substantially improved in last 15 years [5]. With the sustained improvement of outcomes with new agents, there has been a topic of debate about the value of ASCT in the MM treatment modalities. However, the findings of recent large-scale RCTs still support the incorporation of ASCT into the MM treatment process [6, 7]. ASCT as a frontline treatment remains the backbone in the therapy of patients with MM in the current era of novel agents [8].

Successful stem cell mobilization and adequate collection of peripheral blood stem cells (PBSCs) are essential for patients with MM undergoing ASCT. Presently, the mobilization protocols used routinely in clinical practice comprise cytokines, chemo-mobilization, and the CXCR4 inhibitor plerixafor [9]. Cyclophosphamide (CTX) combined with granulocyte-colony stimulating factor (G-CSF) or G-CSF alone are typical regimens for PBSC harvesting. The protocols with CTX plus G-CSF, which have been applied more than 25 years [10] while being efficient, are noted to be associated with serious treatment-related adverse effects, like neutropenic fever and hematuria [11, 12].To reduce the chemotherapeutic toxicity during mobilization, the strategy with G-CSF alone has been introduced [12]. Indeed, several types of research with small sample sizes have compared the effects of the two mobilization regimens but the conclusions still have controversies between studies [13–16]. Whether a contradiction in these data was owing to insufficient sample size or genuine heterogeneity remains unknown. Therefore, we conducted a meta-analysis to compare CTX plus G-CSF and G-CSF alone strategies in terms of the efficiency, safety of mobilization, and survival outcomes after ASCT.

## Methods

### Search strategy

The guidelines of the Preferred Reporting Items for Systematic Reviews and Meta-analyses [17] were followed in our study. We systematically researched the studies published in four databases, PubMed, EMBASE, the Cochrane Library, and Web of Science, up to August 2020 by two independent authors. The search process was performed adopting medical subject headings (MeSH) terms, specific keywords restricted with title or abstract, and combined using the Boolean operators “AND” and “OR”; search terms were appropriately adjusted for different databases. Search details can be found in the Supplementary information file 1.

### Criteria for including and excluding studies

All prospective or retrospective studies investigated, the PBSC mobilization with CTX plus G-CSF and G-CSF alone in MM, were eligible, a detailed description of mobilization regimens was required in all included studies. We excluded the studies as follows: (1) Granulocyte-macrophage colony-stimulating factor (GM-CSF) was combined with CTX or used alone as mobilization regimens; (2) the plerixafor was used in initial mobilization; (3) the meta-analysis, case reports, and reviews were also excluded.

### Literature screen

The de-duplicated bibliography was scanned independently by two authors to exclude apparent unrelated studies. Then, the full text was reviewed, and data were extracted independently by two authors. Controversial opinions were resolved by discussion.

### Data collection and quality assessment

Excel was designed to collect data including the characteristics of the studies, all parameters and values evaluating the efficiency and safety of the two specified mobilization regimens, and survival outcomes after ASCT. Also, the indirectly reported survival data from the Kaplan-Meier curve were obtained by using the Engauge Digitizer software. Following data extraction, the quality of the included studies was assessed by two authors independently. The Cochrane Collaboration’s risk-of-bias tool [18] was adopted for RCTs, and the Newcastle–Ottawa Scale (NOS) tool [19] was used for nonrandomized studies.

### Data synthesis and analysis

The results of the analysis were presented as standard mean differences (SMD), odds ratios (ORs), hazard ratios (HRs), and 95% confidence intervals (CIs). For some continuous variables with medians and quartiles or extreme values, the means and standard deviations (SD) were estimated using previously published methods [20–22]. HRs from the Kaplan-Meier curves were estimated according to Tierney’s approach [23] and for pooling, and the natural logarithm of median survival time ratio (MSR) was used for data processing and as an effect size for median survival data. The meta-analysis was conducted in the R. The test of Cochran’s *Q* and Higgin’s and Thompson’s *I*^2^ [24] was adopted to assess heterogeneity; a fixed-effects model [25] was applied when there was no significant heterogeneity (*I*^2^ < 50% or *p* > 0.1); otherwise, a random-effects model [26] was used. Besides, subgroup analysis was conducted for exploring heterogeneity, and the sensitivity analysis was also performed. The Hartung-Knapp-Sidik-Jonkman (HKSJ) [27–29] method was adopted in the random-effects model for sensitivity analysis. Publication bias was evaluated by Egger’s test [30] when the overall effect pooled more than 10 data sets, and the funnel plot was also displayed. If publication bias was confirmed, the trim-and-fill method developed by Duval and Tweedie [31] was implemented to adjust for bias. All *p* values were 2-sided, and *p* < 0.05 was considered significant.

## Results

### Literature retrieval and screening

**Fig. 1:**
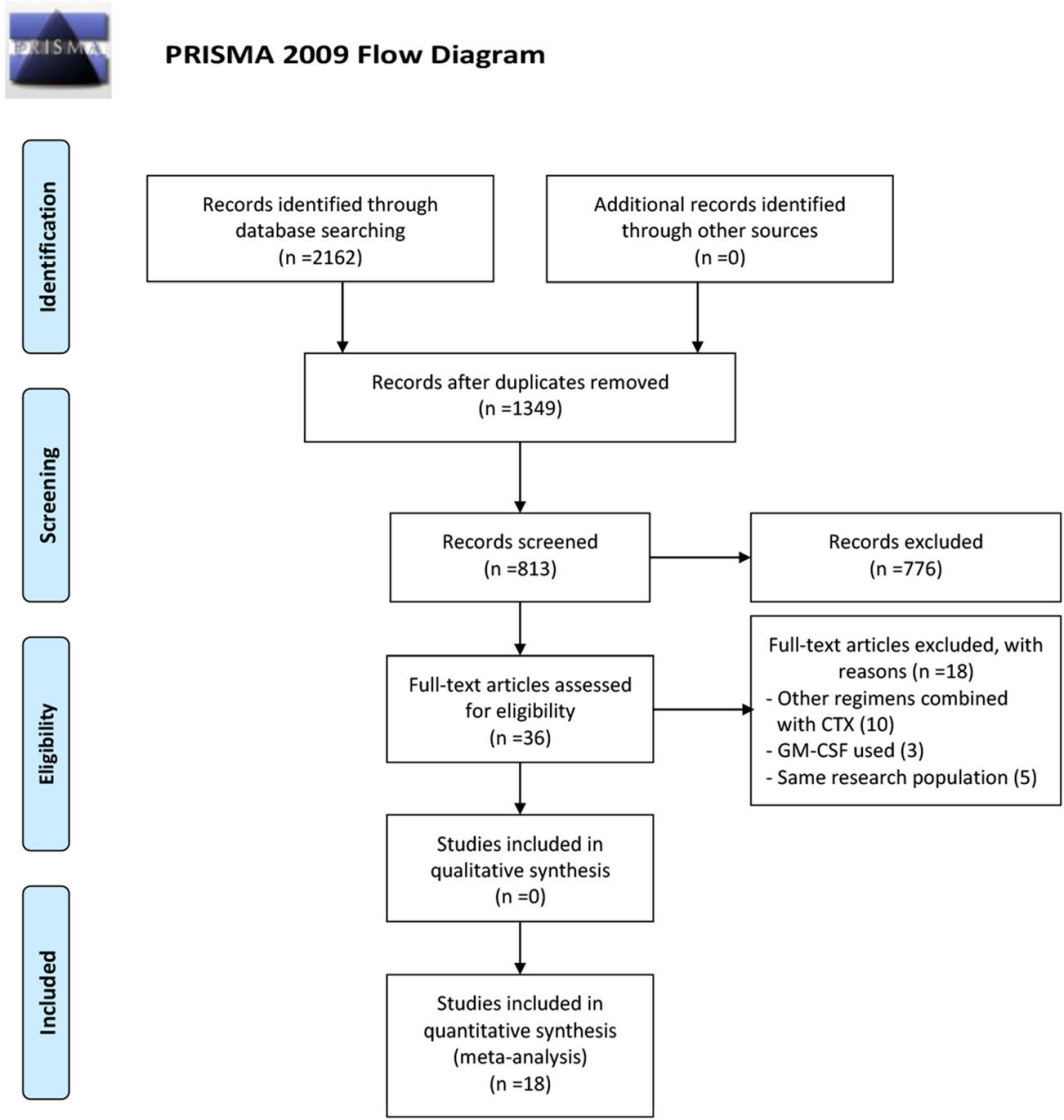
Flowchart of study screening

The initial search retrieved a total of 2162 studies, and 813 studies were excluded due to duplication. After titles and abstracts were previewed, a further 776 irrelevant studies were excluded. Then another 18 studies were excluded after carefully reviewing the full text. Ultimately, a total of 18 studies containing 2770 MM patients met the predefined inclusion criteria. Detailed search procedures are shown in Fig. 1. The characteristics of eligible studies were summarized in Table 1. Among 18 studies, two [15, 32] studies were abstracts presented at the American Society of Hematology (ASH), and one [44] study was a sub-study that shared the same population with another RCT [42]. There are 4 prospective studies including 2 RCTs and 14 retrospective studies. Seven studies were conducted in multiple centers and 11 studies were in single centers. The dose range of CTX was 3–4 g/m^2^ in 10 studies and 1–2 g/m^2^ in 6 studies, one [12] study used the 6 g/m^2^ CTX, and one [34] study reported 2 different CTX dose data sets. The most common dose of G-CSF was 10 μg/kg with filgrastim or lenograstim. One study used RD as induction treatment, and 8 studies adopted exclusive triplet regimens, including 2 VCD, 3 CTD, 1 BiRD, and 2 RVD. The induction regimens were variant in 6 studies, and 3 studies did not report the information. Additionally, 9 studies, including 969 patients who underwent ASCT after mobilization, had reported the survival outcomes between the two mobilization regimens; the features were summarized in Supplementary Table 1.

**Table 1:** Summary characteristics of including studies

Study	Accrual period	Country	Study design	Number		Median (range) age		Gender (Male/Female)		Induction treatment	Mobilization regimens		Quality ^**a**^
				CTX + G-CSF	G-CSF	CTX+ G-CSF	G-CSF	CTX + G-CSF	G-CSF		CTX + G-CSF	G-CSF	
Bacon, W. A. 2011 [32]	2000.12–2008	USA	Retrospective, S	103	83	-	-	-	-	-	CTX 4 g/m^2^ + G-CSF 10 μg/kg	G-CSF 10 μg/kg	b
Benyamini, N. 2017 [33]	2009–2013	Israel	Retrospective, S	56	36	57.3 (35–68)	59.3 (41–74)	39/17	20/16	VCD	CTX 3 g/m^2^ + G-CSF 10 μg/kg	G-CSF 10 μg/kg	8
Chua, C. C. 2018 [34]	2012.12–2015.3	Australia	Retrospective, M	113	175	60 (29–75)	62 (36–71)	74/39	109/66	VCD	CTX 1.5–2 or 3–4 g/m^2^ + filgrastim 5 or 10 μg/kg or pegfilgrastim 12 mg	Filgrastim 10 or 20 μg/kg	8
Crusoe, E. Q. 2016 [16]	2009.5–2013.6	Brazil	Retrospective, M	18	70	56.5 (42–68)	58 (36–69)	13/5	39/31	CTD	CTX 1–2 g/m^2^ + filgrastim 10–15 μg/kg	Filgrastim 15-20 μg/kg	8
de la Rubia J. 2006 [35]	2000.1–2004.12	Spain	Retrospective, M	206	551	59 (29–72)		430/359		ViBMeCP, VBAdD	CTX 1.5 g/m^2^ + G-CSF 5 μg/kg	G-CSF 10–12 or 16-24 μg/kg	8
Desikan, K. R. 1998 [12]	1994.6–1995.7	USA	Prospective, S	22	22	-		-		-	CTX 6 g/m^2^ + G-CSF 5 μg/kg	G-CSF 16 μg/kg	7
Jang, J. E. 2016 [36]	2006.9–2013.8	Korea	Retrospective, M	117	62	53.5 (48–60)	55.5 (48–61)	70/47	36/26	Me, T, R, TR, ViAD	CTX 3 g/m^2^ + G-CSF 10 μg/kg	G-CSF 10 μg/kg	8
Jung, S. H. 2013 [37]	2004.1–2011.10	Korea	Retrospective, S	48	6	55 (39–69)		29/25		CTD	CTX 3 g/m^2^ + G-CSF 10 μg/kg	G-CSF 10 μg/kg	8
Kumar, S. 2007 [38]	2002.1–2006.12	USA	Retrospective, S	134	242	59 (33–75)	59.3 (33–75)	83/51	141/101	D, ViAD, TD, RD	CTX 3 g/m^2^ + filgrastim 10 μg/kg	Filgrastim 10 μg/kg	8
Lin, T. L. 2016 [39]	2003.1–2012.12	China	Retrospective, S	78	10	52.3^**d**^	50.5^**d**^	37/39	7/3	ViAD, TD, VTD, VCD	CTX 2 g/m^2^ + G-CSF 10 μg/kg	G-CSF 10 μg/kg	8
Mark, T. 2008 [40]	2004.12–2007.4	USA	Retrospective, S	20	9	56.3^**d**^	62.3^**d**^	11/8	6/3	BiRD	CTX 3 g/m^2^ + G-CSF 10 μg/kg	G-CSF 10 μg/kg	8
Nakasone, H. 2009 [41]	2000.4–2007.12	Japan	Retrospective, M	67	21	54 (29–66)	57 (46–62)	37/30	13/8	–	CTX 1-2 g/m^2^ + 400 μg/m^2^ filgrastim or 10 μg/kg lenograstim	400 μg/m^2^ filgrastim or 10 μg/kg lenograstim	8
Silvennoinen, R. 2016 [42]	2013.1–2015.2	Finland	RCT, M	34	35	62 (48–69)	63 (40–70)	18/16	19/16	RVD	CTX 2 g/m^2^ + filgrastim 5 μg/kg	Filgrastim 10 μg/kg	Moderate ^**c**^
Skerget, M. 2016 [43]	NA	Slovenia	Prospective, S	9	20	59 (42–63)	60 (35–69)	5/4	11/9	VD	CTX 4 g/m^2^ + filgrastim 10 μg/kg	Filgrastim 10 μg/kg	8
Tanimura, A. 2018 [13]	1999.1–2010.12	Japan	Retrospective, S	115	32	55.1^**d**^	58.3^**d**^	56/69	19/13	ViAD, D, R, T	CTX 4 g/m^2^ + 400 μg/m^2^ filgrastim or 10 μg/kg lenograstim	400 μg/m^2^ filgrastim or 10 μg/kg lenograstim	8
Tuchman, S. A. 2015 [14]	2001–2008	USA	Retrospective, S	94	73	59 (34–76)	57 (33–73)	59/35	29/44	T, R, V	CTX 3–4 g/m^2^ + filgrastim 10 μg/kg	Filgrastim 10 μg/kg	7
Valtola, J. 2016 [44]	2013.1–2014.10	Finland	RCT, M	17	19	58 (49–70)	63 (52–70)	10/7	8/11	RVD	CTX 2 g/m^2^ + filgrastim 5 μg/kg	Filgrastim 10 μg/kg	Moderate ^c^
Whitmill, R. S. 2015 [15]	2003–2010	UK	Retrospective, S	44	45	58 (41–74)	58 (38–70)	55/34		CTD	CTX 3 g/m^2^ + lenogastrim 10 μg/kg	Lenogastrim 10 μg/kg	b

*CTX* & *C*, cyclophosphamide; *G-CSF*, granulocyte-colony stimulating factor; *M*, multiple centers; *S*, single centers; *V*, bortezomib; *T*, thalidomide; *D*, dexamethasone; *Me*, melphalan; *R*, lenalidomide; *Vi*, vincristine; *B*, carmustine; *P*, prednisone; *Ad*, adriamycin; *A*, doxorubicin; *Bi*, clarithromycin

^a^Method of quality assessment based on the study design which described in the “Methods” section

^b^Abstract of meeting

^c^Due to risk of blindness

^d^Mean age

### Mobilization efficiency

**Fig. 2:**
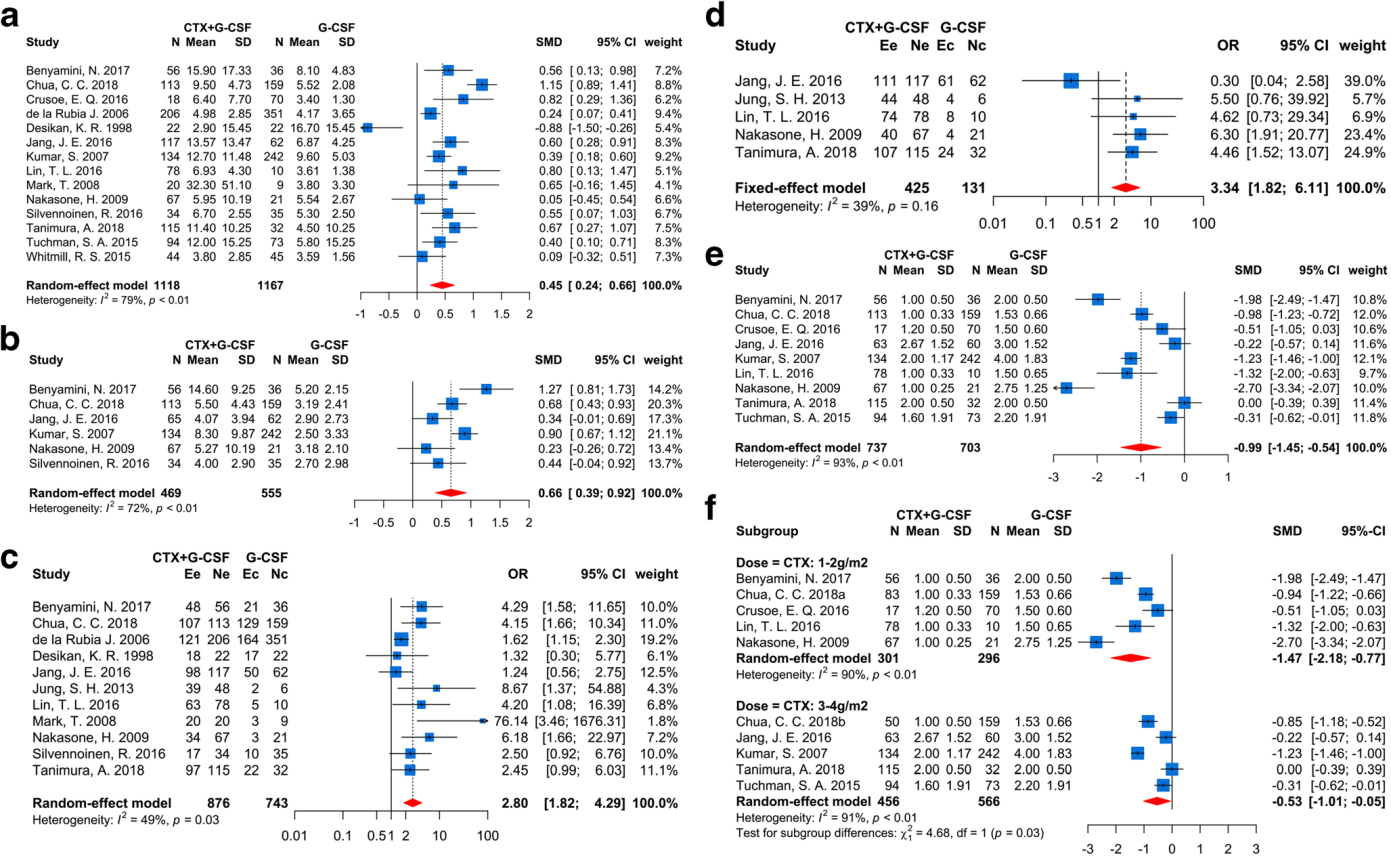
Forest plots of mobilization efficiency between CTX plus G-CSF and G-CSF alone regimens. **a** Total CD34^+^ cells collection. **b** CD34^+^ cells amount collected on the first day. **c** Rate of collection ⩾ 4 × 10^6^/kg CD34^+^ cells. **d** Rate of collection ⩾ 2 × 10^6^/kg CD34^+^ cells. **e** Apheresis times during mobilization. **f** Subgroup analysis–based CTX dose for apheresis times during mobilization

Fourteen studies [12–16, 33–36, 38–42] including 2285 patients reported the total CD34^+^ cells (10^6^/kg) yield between CTX plus G-CSF and G-CSF alone mobilization regimens. Due to significant heterogeneity tested (*I*^2^ = 79.3%, *p* < 0.0001), a random-effects model was adopted, and the results showed that CTX plus G-CSF regimens yield more CD34^+^ cells than G-CSF alone (SMD = 0.45, 95% CI (0.24, 0.66), *p* < 0.0001, Fig. 2a). In addition, CTX subgroup analysis was also performed with random-effects model (Supplementary Fig. 1a), while the pooled effects between 3 and 4 g/m^2^ and 1–2 g/m^2^ group had no difference (*p* = 0.84). The different dose of CTX used was not the source of heterogeneity between studies. Besides, CD34+ cells amount collected on the first day was higher in CTX plus G-CSF group than that in G-CSF-alone group to a limit degree (*I*^2^ = 71.6%, SMD = 0.66, 95% CI (0.39, 0.92), *p* < 0.0001, Fig. 2b). Similarly, high-dose CTX treatment revealed an undifferentiated benefit compared to the low dose (SMD = 0.71 and 0.66, respectively, *p* = 0.82, Supplementary Fig. 1b).

**Fig. 3:**
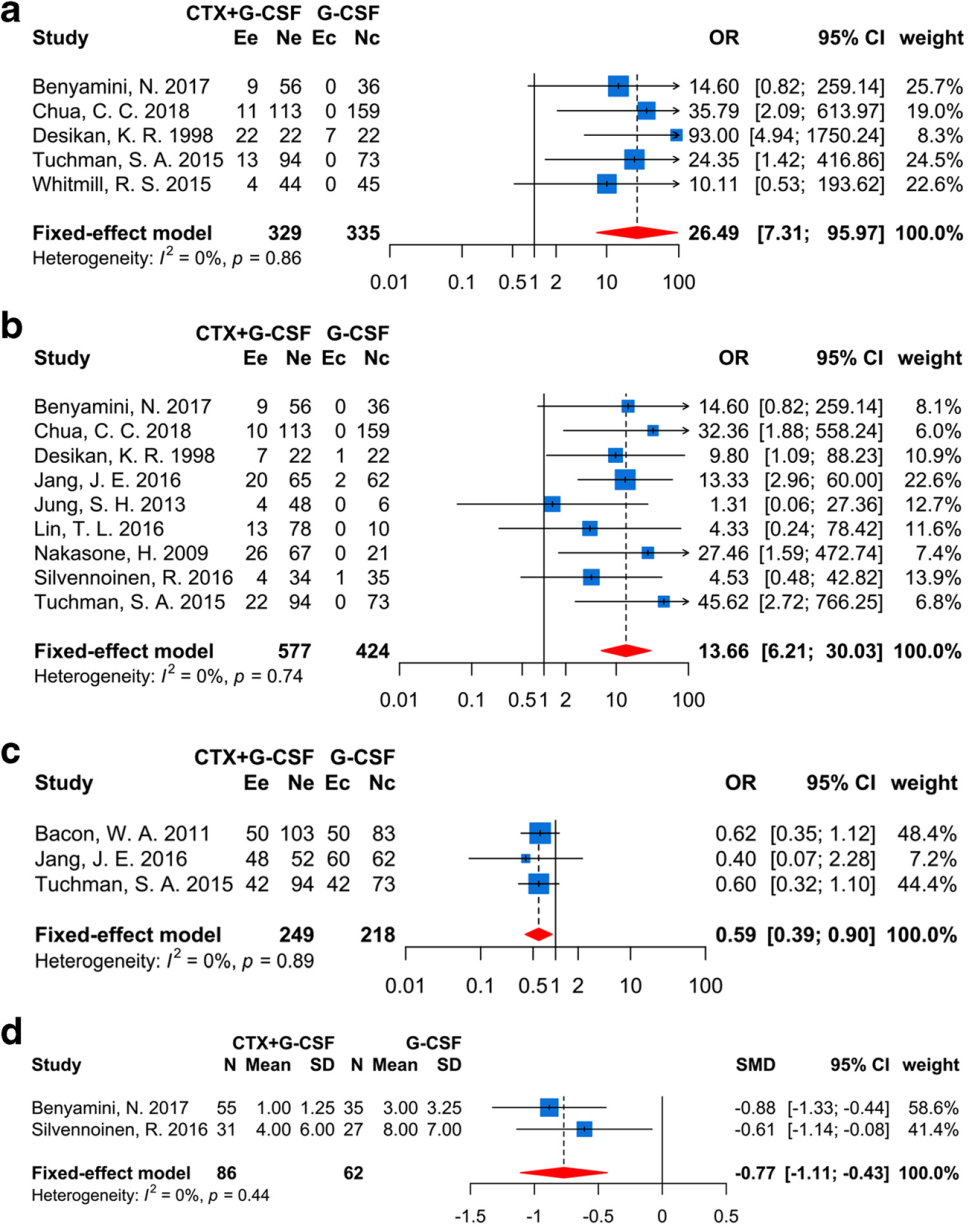
Forest plots of safety during mobilization and response after ASCT between CTX plus G-CSF and G-CSF-alone regimens. **a** Admission rate during mobilization. **b** Fever rate during mobilization. **c** Response to VGPR or better after ASCT. **d** Units of platelet infusion needed during ASCT

In general, a minimal CD34^+^ cells target to undergo one ASCT was ⩾ 2 × 10^6^/kg, successful mobilization usually defined as collection ⩾ 4 × 10^6^/kg CD34^+^ cells considering two ASCTs [45]. Eleven studies [12, 13, 33–37, 39–42] with 1619 patients included in the meta-analysis had compared the rate of collection ⩾ 4 × 10^6^/kg CD34^+^ cells between the two mobilization regimens; the random-effects model (heterogeneity: *p* = 0.03) showed that the CTX plus G-CSF group had 2.8-fold higher successful mobilization rate than G-CSF alone (OR = 2.8, 95% CI (1.82, 4.29), *p* < 0.0001, Fig. 2c). As regards to the rates of minimal target ⩾ 2 × 10^6^/kg CD34^+^ cells, the pooled effect also displayed an obvious advantage in the CTX plus G-CSF group (*I*^2^ = 39%, OR = 3.34, 95% CI (1.82, 6.11), *p* < 0.0001, Fig. 2d). For subgroup analysis, different doses of CTX administration showed similar effects in both successful (Supplementary Fig. 1d) and minimal (Supplementary Fig. 1c) CD34+ cell mobilization (*p* = 0.61 and 0.34, respectively). Additionally, apheresis times during mobilization were detected smaller in the patients who received CTX plus G-CSF regimens (*I*^2^ = 90.7%, SMD = − 0.80, 95% CI (− 1.21, − 0.38), *p* = 0.0002, Fig. 2e). Of note, a low dose of CTX with 1–2 g/m^2^ displayed a more significant reduction of apheresis times than the 3–4 g/m^2^ group (SMD = − 1.47 and − 0.53, respectively, *p* = 0.03, Fig. 3f).

### Safety of mobilization

Certainly, 5 studies [12, 14, 15, 33, 34] had coherent tendency (heterogeneity: *I*^2^ = 0%, *p* = 0.86) that CTX plus G-CSF administration demonstrated a higher risk of admission rate than G-CSF alone during mobilization (OR = 26.49, 95% CI (7.31, 95.97), *p* < 0.0001, Fig. 3a). Similarly, the fever rate was also higher in the CTX plus G-CSF group (OR = 13.66, 95% CI (6.21, 30.03), *p* < 0.0001, Fig. 3b), according to a fixed-effects model (*I*^2^ = 0%, *p* = 0.74) including 9 studies [12, 14, 33, 34, 36, 37, 39, 41, 42] and 999 MM patients. Moreover, two doses of CTX showed an undifferentiated effect to fever risk in subgroup analysis (*p* = 0.58, Supplementary Fig. 1e).

### Response and adverse effects during ASCT

In the present study, 9 studies (Supplementary Table 1), including 969 patients, were processed to ASCT after mobilization. With regard to the response of patients after ASCT, the proportion of patients who attained very good partial response (VGPR) or better in CTX plus G-CSF (56.2%) group was lower than the G-CSF-alone (69.7%) group (*I*^2^ = 0%, OR = 0.59, 95% CI (0.39, 0.90), *p* = 0.01, Fig. 3c). However, the complete response (CR) rate and VGPR rate had no difference between the two groups (*p* = 0.11 (Supplementary Fig. 2a) and *p* = 0.98 (Supplementary Fig. 2b), respectively). About the neutrophil and platelet engraftment, the days of neutrophil recovery to 0.5 × 10^9^/L and platelet recovery to 20 × 10^9^/L after ASCT were similar between the two mobilization regimens (*p* = 0.99 (Supplementary Fig. 2c) and 0.96 (Supplementary Fig. 2d), respectively. Besides, fewer units of platelet infusion were needed for patients during ASCT who used CTX plus G-CSF mobilization protocols *(I*^2^ = 0%, SMD = − 0.77, 95% CI (− 1.11, − 0.43), *p* < 0.0001, Fig. 3d). There had no difference about the treatment-related mortality, infusion of red blood cells, days in hospital, rates of fever, and pneumonitis during ASCT between the two regimens, the lymphocyte (10^9^/L) recovery on day 15 after ASCT (*p* = 0.26 (Supplementary Fig. 2e), 0.3 (Supplementary Fig. 2f), 0.72 (Supplementary Fig. 2g), 0.07 (Supplementary Fig. 2h), 0.87 (Supplementary Fig. 2i), and 0.14 (Supplementary Fig. 2j), respectively.

### Survival outcomes after ASCT

**Fig. 4:**
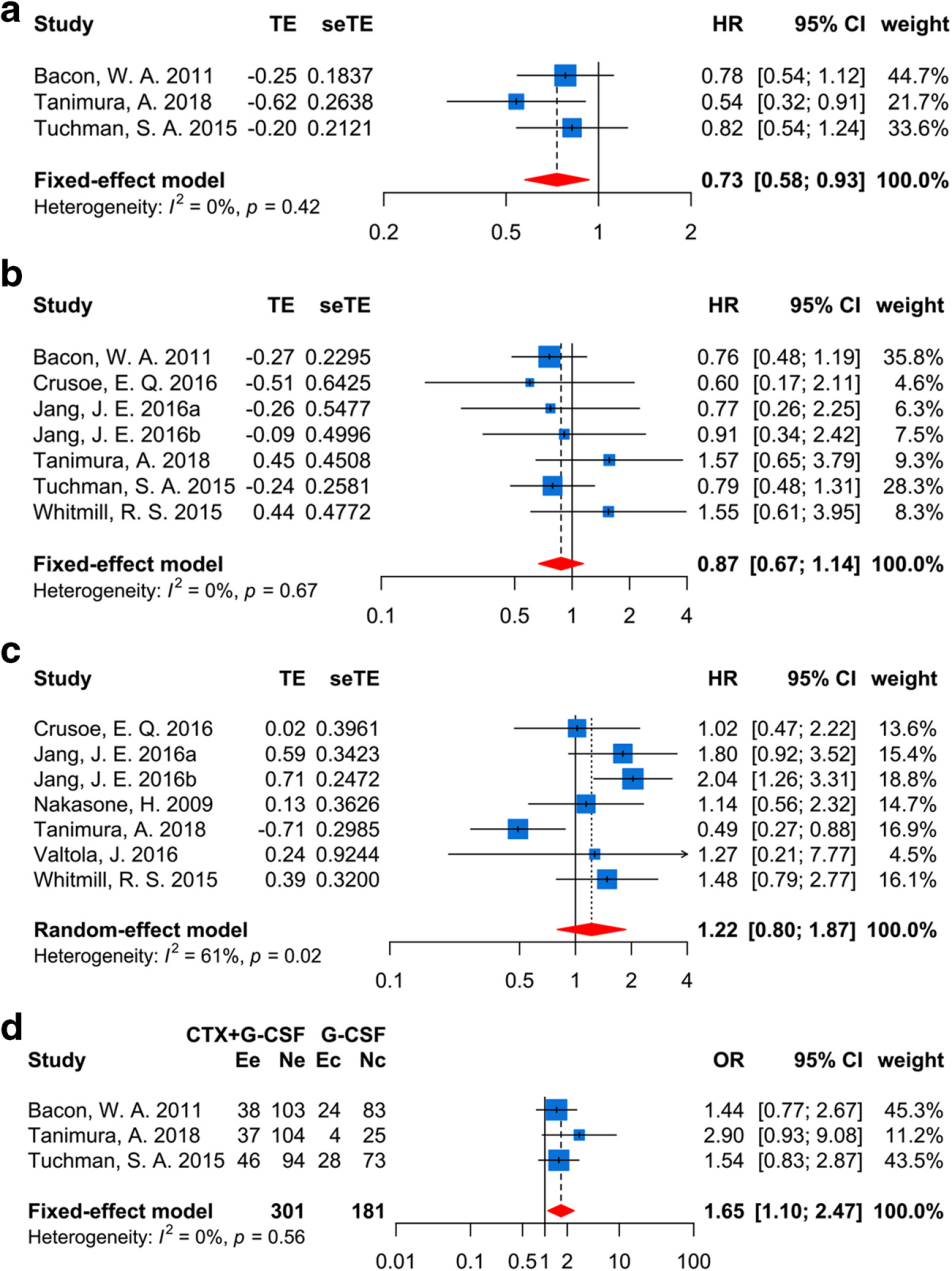
Survival outcomes of the patients who mobilized with CTX plus G-CSF or G-CSF alone regimens. **a** Event-free survival (EFS) with univariate data. **b** Overall survival (OS) with univariate data. **c** Progression-free survival (PFS) with univariate data. **d** 3-year EFS rate

There were three different survival endpoints reported in the included studies; the overall survival (OS), the progression-free survival (PFS), and the event-free survival (EFS) based on univariate and multivariate analysis were computed respectively. Pooled EFS without heterogeneity (*I*^2^ = 0%) based on 3 univariate data sets [13, 14, 32] showed patients mobilized with CTX plus G-CSF had a better EFS (*I*^2^ = 0%, HR = 0.73, 95% CI (0.56, 0.93), *p* = 0.01, Fig. 4a). However, no decisively significant tested in multivariate data (HR = 0.7, *p* = 0.45, Supplementary Fig. 3a). Notably, patients who underwent different mobilization regimens shared an equivalent OS in the meta-analysis (univariate: *I*^2^ = 0%, HR = 0.87, *p* = 0.33, Fig. 4b; multivariate: *I*^2^ = 0%, HR = 0.89, *p* = 0.64, Supplementary Fig. 3b). Similar conclusions were drawn in PFS (univariate: *I*^2^ = 61.3%, HR = 1.22, *p* = 0.36, Fig. 4c; multivariate: *I*^2^ = 51.9%, HR = 0.57, *p* = 0.13, Supplementary Fig. 3c).

For the median survival time, computed MSR displayed that no significant difference was detected in the two groups about median OS, EFS, and PFS time (*p* = 0.97 (Supplementary Fig. 3d), 0.45 (Supplementary Fig. 3e), and 0.91 (Supplementary Fig. 3f), respectively). The probability of OS and PFS at 1, 3, and 5 years among patients who underwent different mobilizations were consistent (Supplementary Fig. 3g–l), but the patients in the CTX plus G-CSF group (40.2%) had a higher 3-year EFS rate than that in the G-CSF-alone (30.1%) group (*I*^2^ = 0%, OR = 1.65, 95% CI (1.1, 2.47), *p* = 0.02, Fig. 4d). However, combined 1-year and 5-year EFS rates were equivalent (*p* = 0.12 (Supplementary Fig. 3m) and 0.39 (Supplementary Fig. 3n), respectively).

### Sensitivity analysis and publication bias

The results of the sensitive analysis using different models are summarized in Table 2 and Supplementary Table 2; all pooled results with statistical significance were stable. Meanwhile, the forest plots recalculated the pooled effects with one study omitted each time were generated (Supplementary Figs. 4 and 5). The publication bias was only tested in two comparisons due to enough studies included (above 10). No publication bias detected in the comparisons of total CD34^+^ cells collection (*p* = 0.99). For the comparison of successful mobilization rate in the meta-analysis, a significant publication bias detected (*p* = 0.006), but the relationship was unaffected (OR = 2.05, 95% CI (1.31, 3.21), *p* = 0.002) when reanalyzed by adopting the trim-and-fill [31] method as described previously. The funnel plots for the two comparisons are displayed in Supplementary Fig. 6.

**Table 2:** Summary results of sensitivity analysis

Parameters	Participants in analysis	Effect sizes ^*^	M-H fixed-model (effect, 95% CI)	D-L random-model (effect, 95% CI)	HKSJ random-model (effect, 95% CI)	Heterogeneity	*p* value ^**a**^
EFS-U	482	HR	*0.73 (0.56, 0.93)* ^*b*^	0.73 (0.58, 0.93)	0.72 (0.43, 1.21)	*I*^2^: 0% *p* = 0.4	*p* = 0.01
3-year EFS	482	OR	*1.65 (1.10, 2.47)*	1.62 (1.08, 2.44)	1.65 (0.78, 3.48)	*I*^2^: 0% *p* = 0.56	*p* = 0.02
Total CD34^+^ cells collection (10^6^/kg)	2285	SMD	0.45 (0.37, 0.54)	*0.45 (0.24, 0.66)*	0.45 (0.18, 0.71)	*I*^2^: 79.3% *p* < 0.0001	*p* < 0.0001
CD34^+^ cells collection on first day (10^6^/kg)	1024	SMD	0.71 (0.58, 0.64)	*0.66 (0.39, 0.92)*	0.65 (0.26, 1.05)	*I*^2^: 71.6% *p* = 0.004	*p* < 0.0001
Collection ⩾ 4 × 10^6^/kg CD34^+^ cells	1619	OR	2.25 (1.77, 2.87)	*2.80 (1.82, 4.29)*	3.09 (1.75, 5.44)	*I*^2^: 48.9% *p* = 0.03	*p* < 0.0001
Collection ⩾ 2 × 10^6^/kg CD34^+^ cells	556	OR	*3.34 (1.82, 6.11)*	3.58 (1.48, 8.69)	3.38 (0.82, 13.97)	*I*^2^: 39.0% *p* = 0.16	*p* < 0.0001
Days of apheresis	1352	SMD	− 0.81 (− 0.93, − 0.69)	*− 0.80 (− 1.21, − 0.38)*	− 0.80 (− 1.36, − 0.24)	*I*^2^: 90.7% *p* < 0.0001	*p* = 0.0002
Rate of admission during mobilization	664	OR	*26.49 (7.31, 95.97)*	26.06 (7.16, 94.81)	26.05 (9.09, 74.69)	*I*^2^: 0% *p* = 0.86	*p* < 0.0001
Rate of fever during mobilization	999	OR	*13.66 (6.21, 30.03)*	11.02 (4.96, 24.48)	10.92 (5.01, 23.78)	*I*^2^: 0% *p* = 0.74	*p* < 0.0001
Response to VGPR or better after ASCT	467	OR	*0.59 (0.39, 0.90)*	0.60 (0.39, 0.90)	0.60 (0.44, 0.81)	*I*^2^: 0% *p* = 0.89	*p* = 0.01
Units of platelet infusions during ASCT	148	SMD	*− 0.77 (− 1.11, − 0.43)*	− 0.77 (− 1.11, − 0.43)	− 0.77 (− 2.49, 0.96)	*I*^2^: 0% *p* = 0.44	*p* < 0.0001

*M-H*, Mantel-Haenszel statistical method for fixed-effects model; *D-L*, DerSimonian-Laird statistical method for random effects model, *HKSJ*, Hartung-Knapp-Sidik-Jonkman adjustment for random effects model; *EFS*, event-free survival; *U*, univariate; *HR*, hazard ratio; *OR*, odds ratio; *SMD*, standard mean difference; *ASCT*, autologous stem cell transplantation; *VGPR*, very good partial response

*All effect sizes were calculated by comparing the CTX plus G-CSF to G-CSF alone

^a^The *p* value of test for overall effect

^b^Values in italics are the estimated overall effect in this study

## Discussion

Generally, one big challenge for MM patients is the relapse; after each relapse, the disease will become more aggressive with shortened subsequent PFS [46]. Collections of autologous stem cells are often contaminated with myeloma cells, which might make a disputable contribution to the relapse of the disease [47]. To facilitate more CD34^+^ cells yield and additional anti-myeloma effects, CTX was combined with hematopoietic growth factors (like G-CSF or GM-CSF) as a common regimen for PBSC mobilization. The dual functions of CTX might translate into a more effective mobilization and better disease control in MM patients [13, 34]. There are several retrospective studies that have discussed the clinical benefits and risks if CTX is administrated during mobilization; the conclusions still have arguments [48]. Additionally, only one well-designed RCT [42] with small cases has compared the CTX plus G-CSF and G-CSF-alone regimens in MM, which demonstrated that G-CSF alone was successful in most of patients to attain the defined collection target, and no difference in PFS between the study arms [44].

To the best of our knowledge, this is the first meta-analysis to compare the efficiency, safety, and survival outcomes between the two mobilization regimens for ASCT among patients with MM. As expected, patients who received CTX combined with G-CSF treatment had more effective mobilization, which was reflected by a higher PBSC collection in total and on the first day (*p* < 0.0001), as well as higher mobilization rates of defined PBSC collection target (*p* < 0.0001). However, the risks of admission and fever during mobilization were also increased accordingly (*p* < 0.0001). Posttransplant survival outcomes in MM patients who underwent CTX plus G-CSF and G-CSF-alone regimen mobilization were investigated in several studies. Tanimura et al. [13] reported an improved PFS and EFS in patients who adopted the CTX plus G-CSF regimens, although some trials have indicated otherwise [14, 32, 44]. The pooled results in our meta-analysis also showed a favorable EFS (HR = 0.73, *p* = 0.01) and a better 3-year EFS rate (OR = 1.65, *p* = 0.02) in the CTX plus G-CSF group, which indicated that the CTX plus G-CSF mobilization schedule was advantageous to benefit patients with MM remaining event-free after ASCT. However, there was no difference in OS and PFS between the MM patients who mobilized with different regimens in the meta-analysis. Notably, the dose discrepancy of CTX contributed a negligible effect for the difference according to our subgroup analysis, and the overall post-ASCT toxicity was similar in the two groups. The induction treatment with different agents was reported to have a dissimilar impact on the PBSC harvest [38, 49]; however, due to variant induction therapies used between studies included in our analysis, induction therapy–based subgroup analysis was not performed in the meta-analysis.

The models of mobilization of PBSC in ASCT have evolved in recent years [9]. Plerixafor is a state-of-the-art small-molecule drug that is approved for PBSC mobilization as it selectively blocked the CXCR4 receptor, which participates in the trafficking and homing of stem cells to the bone marrow (BM) [50, 51]. A well-designed RCT had confirmed the obvious advantages of plerixafor for PBSC mobilization in patients with MM [52], even as a salvage agent for typical regimens with previous mobilization failure [42]. More importantly, plerixafor also presents an anti-myeloma effect by inhibiting the MM cells homing back to BM [53]. Foreseeably, plerixafor with G-CSF will be an optimal mobilization strategy in the future. However, the high cost of plerixafor precludes its routine administration in all patients, but it simply plays an on-demand role for typical mobilization protocols [54].

Although we attempted to conduct comprehensively analyzed of these included studies, some shortages and immanent limitations need to be acknowledged. There are only two RCTs with the same population included in our analysis; most of them are retrospective studies. Secondly, some pooled data were estimated from the raw values of publications based on the widely acceptable mathematical methods; it may be a partial source of heterogeneity and bias. More large-scaled RCTs are needed in the future.

## Conclusion

Based on present evidence in our meta-analysis, the CTX plus G-CSF regimen had more advantages in mobilization efficacy, as well as more prolonged EFS in patients with MM after ASCT. Serious adverse effects like treatment-related mortality were consistent, although the risks of admission and fever during mobilization were increased. CTX plus G-CSF regimen was superior to G-CSF-alone regimen for PBSC mobilization in patients with MM.

## Supplementary information

Supplementary Fig. 1 Forest plots of subgroup analysis based CTX dose. **A**: Total CD34^+^ cells collection. **B**: CD34^+^ cells amount collected on the first day. **C**: Rate of collection ⩾ 4x10^6^/kg CD34^+^ cells. **D**: Rate of collection ⩾ 2x10^6^/kg CD34^+^ cells. **E**: Fever rate during mobilization. (JPG 2166 kb)

Supplementary Fig. 2 Forest plots of non-survival data. **A**: Response to CR after ASCT. **B**: Response to VGPR after ASCT. **C**: Days of neutrophil recovery to 0.5 × 10^9^/L after ASCT. **D**: Days of platelet recovery to 20x10^9^/L after ASCT. **E**: Treat-related mortality. **F**: Units of red blood cells infusion needed during ASCT. **G**: Days in hospital during ASCT. **H**: Rate of fever during ASCT. **I**: Rate of pneumonitis during ASCT. **J**: Lymphocytes recovery at day 15 after ASCT (10^9^/L). (JPG 1744 kb)

Supplementary Fig. 3 Forest plots of survival data. **A**: Event-free survival (EFS) with multivariate data. **B**: Overall survival (OS) with multivariate data. **C**: Progression-free survival (EFS) with multivariate data. **D**: Median OS time. **E**: Median EFS time. **F**: Median PFS time. **G**: 1-year OS. **H**: 3-year OS. **I**: 5-year OS. **J**: 1-year PFS. **K**: 3-year PFS. **L**: 5-year PFS. **M**: 1-year EFS. **N**: 5-year EFS. (JPG 2498 kb)

Supplementary Fig. 4 Forest plots of the recalculated pooled effects with one study omitted each time for non-survival data. **A**: Total CD34+ cells collection. **B**: CD34+ cells collection on the first day. **C**: Rate of collection ⩾ 4x10^6^/kg CD34^+^ cells. **D**: Rate of collection ⩾ 2x10^6^/kg CD34^+^ cells. **E**: Apheresis times during mobilization. **F**: Admission rate during mobilization. **G**: Fever rate during mobilization. **H**: Response to VGPR or better after ASCT. **I**: Response to CR after ASCT. **J**: Response to VGPR after ASCT. **K**: Days of neutrophil recovery to 0.5x10^9^/L after ASCT. **L**: Days of platelet recovery to 20x10^9^/L after ASCT. **M**: Lymphocytes recovery at day 15 after ASCT (10^9^/L). **N**: Units of platelet infusion needed during ASCT. **O**: Treat-related mortality. **P**: Units of red blood cells infusion needed during ASCT. **Q**: Days in hospital during ASCT. **R**: Rate of fever during ASCT. **S**: Rate of pneumonitis during ASCT. (JPG 6860 kb)

Supplementary Fig. 5 Forest plots of the recalculated pooled effects with one study omitted each time for survival data. **A**: Overall survival (OS) with univariate data. **B**: OS with multivariate data. **C**: Event-free survival (EFS) with univariate data. **D**: EFS with multivariate data. **E**: Progression-free survival (PFS) with univariate data. **F**: PFS with multivariate data. **G**: Median OS times. **H**: Median EFS times. **I**: Median PFS times. **J**: 1-year OS. **K**: 3-year OS. **L**: 5-year OS. **M**: 1-year PFS. **N**: 3-year PFS. **O**: 5-year PFS. **P**: 1-year EFS. **Q**: 3-year EFS. **R**: 5-year EFS. (JPG 6490 kb)

Supplementary Fig. 6 Funnel plots for publication bias. **A**: Total CD34+ cells collection. **B**: Rate of collection ⩾ 4x10^6^/kg CD34^+^ cells. (JPG 438 kb)

ESM 7 (DOCX 17 kb)

ESM 8 (DOCX 24 kb)

ESM 9 (DOCX 15 kb)

## Data Availability

All supporting data are included in the article and its additional files.
