# Supplementary figures and images for: Comparison of the efficiency, safety, and survival outcomes in two stem cell mobilization regimens with cyclophosphamide plus G-CSF or G-CSF alone in multiple myeloma: a meta-analysis
Source: Ann Hematol. 2021 Jan 6;100(2):563–73. doi: 10.1007/s00277-020-04376-w (PMC7817584; doi:10.1007/s00277-020-04376-w)

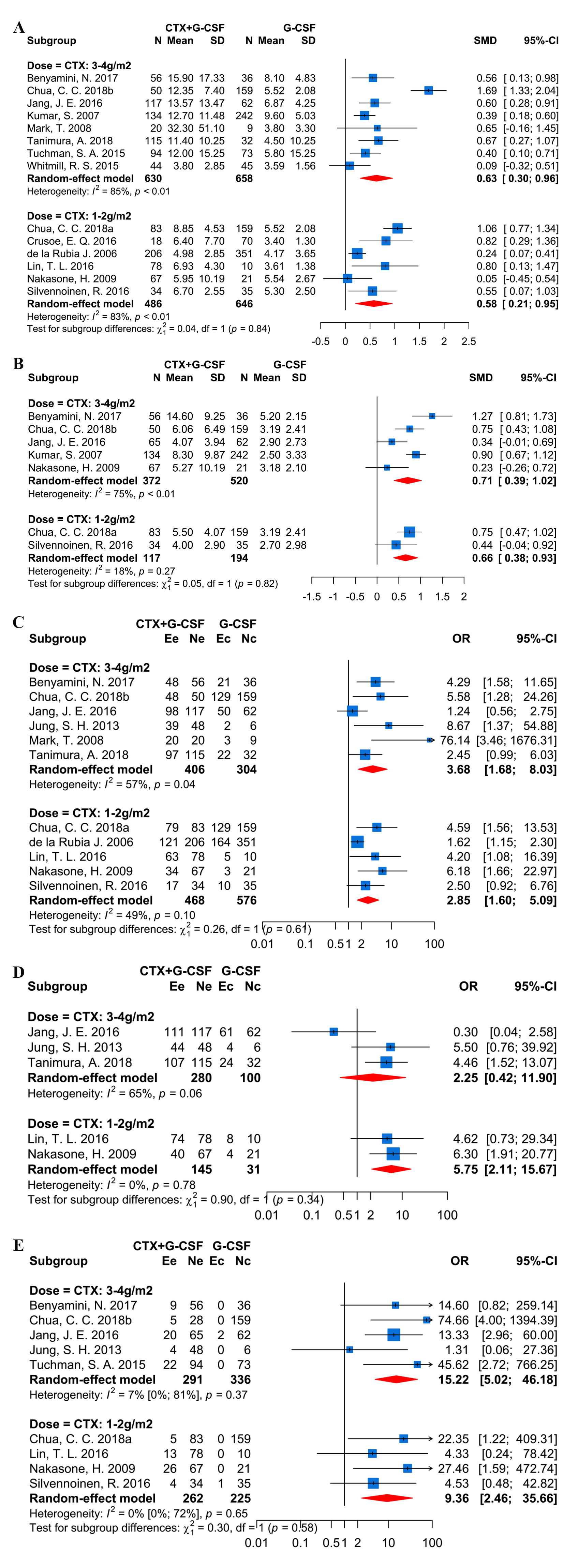

Supplement: Supplementary file 1 — Forest plots of subgroup analysis based CTX dose. A: Total CD34+ cells collection. B: CD34+ cells amount collected on the first day. C: Rate of collection ⩾ 4x10^6^/kg CD34+ cells. D: Rate of collection ⩾ 2x10^6^/kg CD34+ cells. E: Fever rate during mobilization. (JPG 2166 kb) [file 277_2020_4376_MOESM1_ESM.jpg]

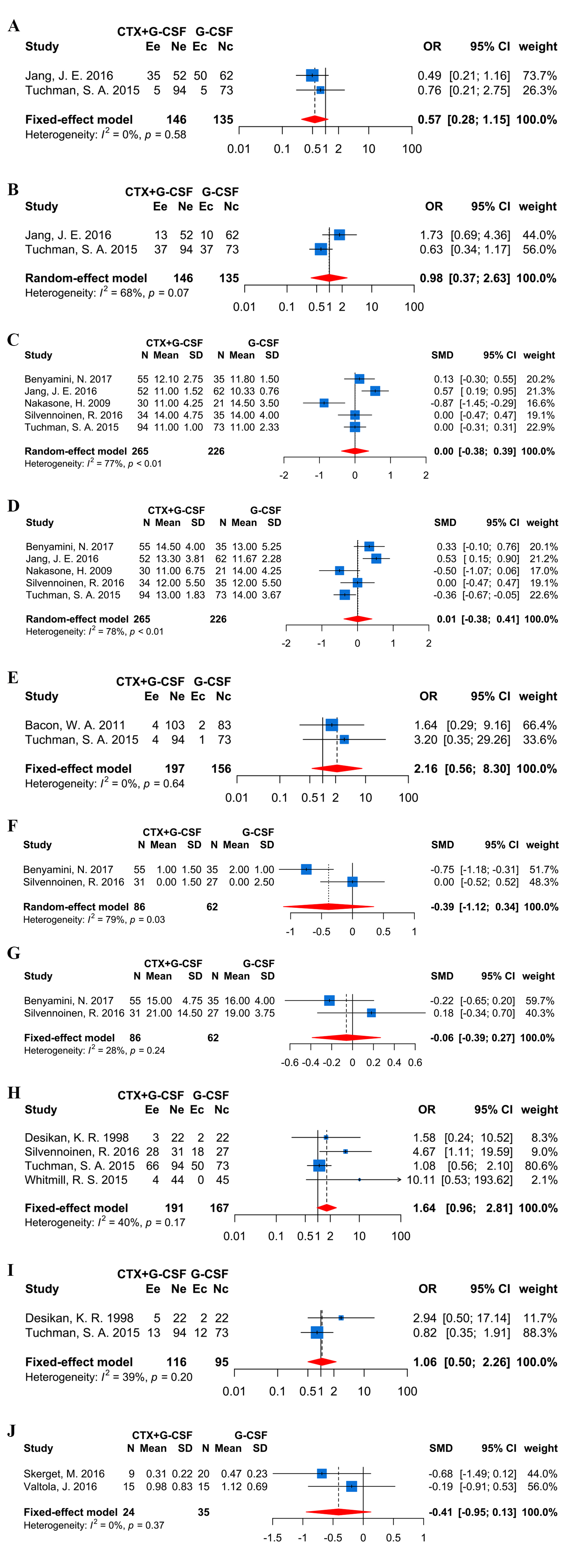

Supplement: Supplementary file 2 — Forest plots of non-survival data. A: Response to CR after ASCT. B: Response to VGPR after ASCT. C: Days of neutrophil recovery to 0.5 × 10^9^/L after ASCT. D: Days of platelet recovery to 20x109/L after ASCT. E: Treat-related mortality. F: Units of red blood cells infusion needed during ASCT. G: Days in hospital during ASCT. H: Rate of fever during ASCT. I: Rate of pneumonitis during ASCT. J: Lymphocytes recovery at day 15 after ASCT (109/L). (JPG 1744 kb) [file 277_2020_4376_MOESM2_ESM.jpg]

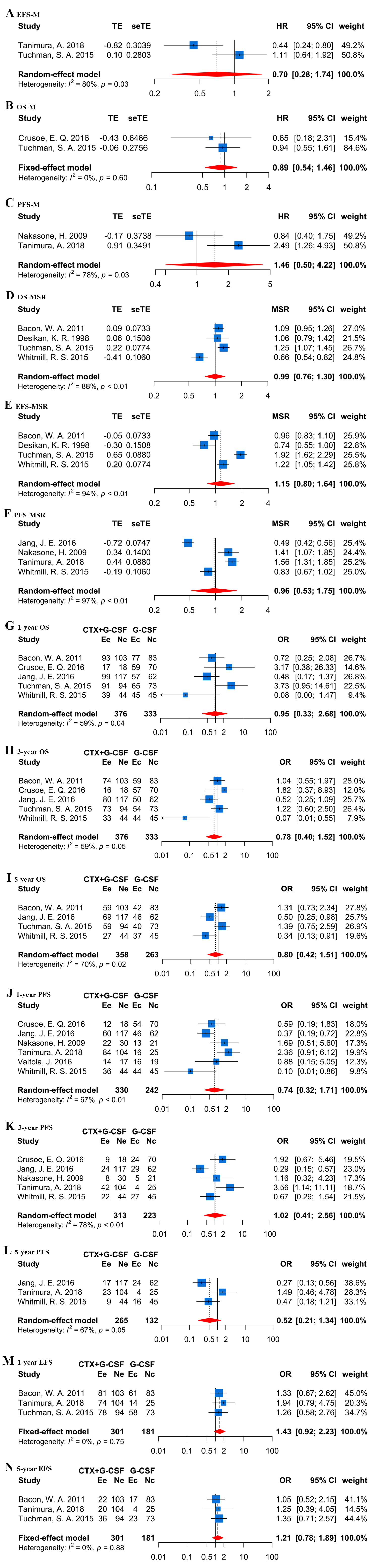

Supplement: Supplementary file 3 — Forest plots of survival data. A: Event-free survival (EFS) with multivariate data. B: Overall survival (OS) with multivariate data. C: Progression-free survival (EFS) with multivariate data. D: Median OS time. E: Median EFS time. F: Median PFS time. G: 1-year OS. H: 3-year OS. I: 5-year OS. J: 1-year PFS. K: 3-year PFS. L: 5-year PFS. M: 1-year EFS. N: 5-year EFS. (JPG 2498 kb) [file 277_2020_4376_MOESM3_ESM.jpg]

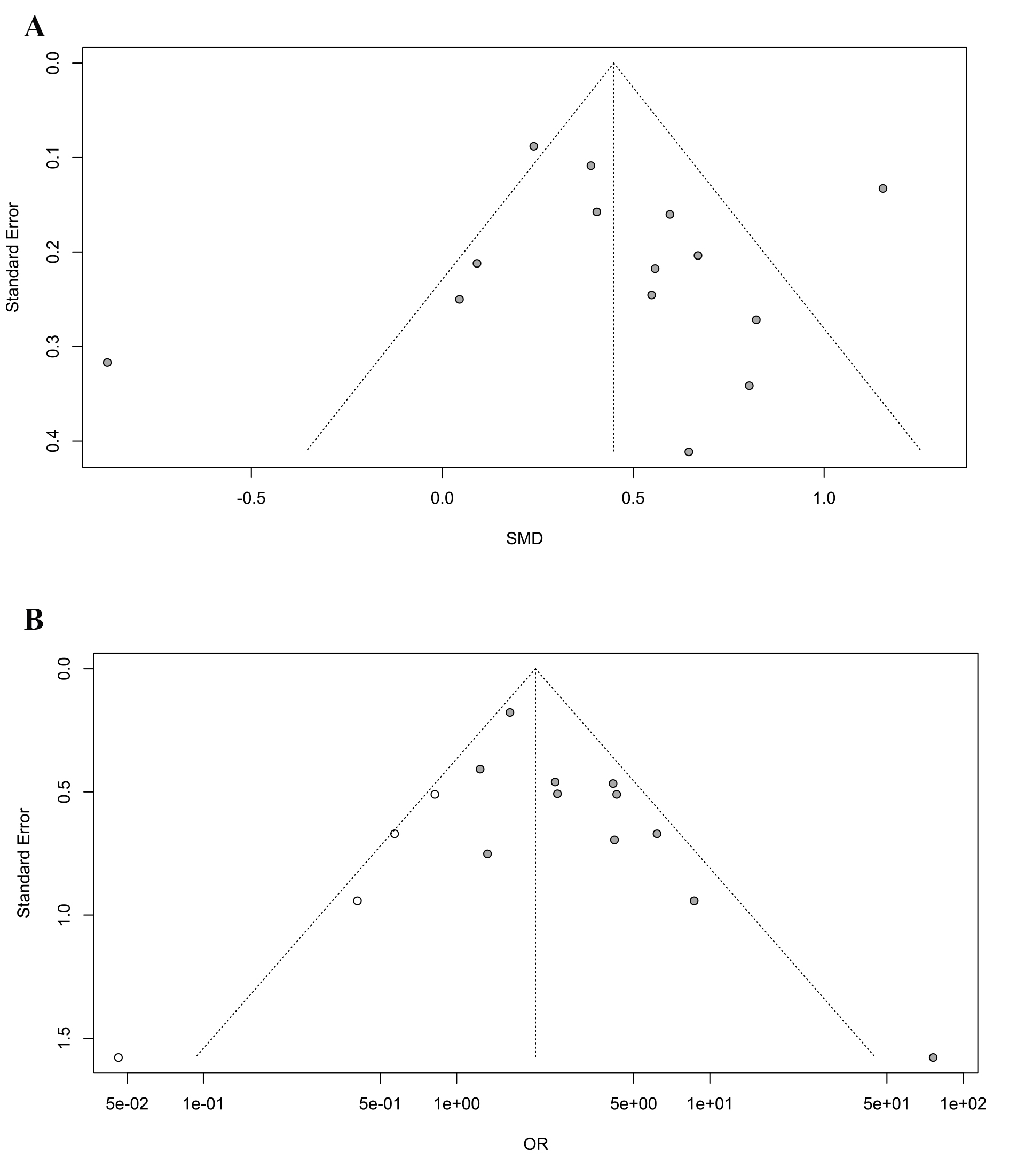

Supplement: Supplementary file 6 — Funnel plots for publication bias. A: Total CD34+ cells collection. B: Rate of collection ⩾ 4x10^6^/kg CD34+ cells. (JPG 438 kb) [file 277_2020_4376_MOESM6_ESM.jpg]
