# Supplementary material for: Comparison of the efficiency, safety, and survival outcomes in two stem cell mobilization regimens with cyclophosphamide plus G-CSF or G-CSF alone in multiple myeloma: a meta-analysis
Source: Ann Hematol. 2021 Jan 6;100(2):563–73. doi: 10.1007/s00277-020-04376-w (PMC7817584; doi:10.1007/s00277-020-04376-w)
Supplement: Supplementary file 7 — (DOCX 17 kb) [file 277_2020_4376_MOESM7_ESM.docx]

| **Supplementary. Table 1. Summary Characteristics of Patients Underwent ASCT** | | | | | | |
| --- | --- | --- | --- | --- | --- | --- |
| Study | Number underwent ASCT | | | Median follow-up (month) | | ASCT procedures |
|  | CTX+  G-CSF | G-CSF | Total | CTX+  G-CSF | G-CSF |  |
| Bacon, W. A. 2011[32] | 103 | 83 | 186 | 34.3 | 32.7 | melphalan 200 mg/m^2^ |
| Crusoe, E. Q. 2016[16] | 18 | 70 | 88 | 28.6 | | - |
| Desikan, K. R. 1998[12] | 22 | 22 | 44 | - | | melphalan 200 mg/m^2^ |
| Jang, J. E. 2016[38] | 117 | 62 | 179 | 30.8 | 34.1 | melphalan 140-200 mg/m^2^ (97%); bortezomib/busulfan/melphalan (3%) |
| Nakasone, H. 2009[43] | 30 | 21 | 51 | 24 | | melphalan 200 mg/m^2^ |
| Tanimura, A. 2018[13] | 104 | 25 | 129 | 58.2 **^a^** | 30 **^a^** | - |
| Tuchman, S. A. 2015[14] | 94 | 73 | 167 | - | | melphalan 140-200 mg/m^2^ |
| Valtola, J. 2016[33] | 17 | 19 | 36 | 15.8 | 14.4 | melphalan 200 mg/m^2^ |
| Whitmill, R. S. 2015[15] | 44 | 45 | 89 | - | | - |
| ***ASCT***: Autologous stem cell transplantation; ***CTX***: Cyclophosphamide; ***G-CSF***: Granulocyte-colony stimulating factor; **^a^** Mean follow-up (month) | | | | | | |
