# Supplementary material for: Comparison of the efficiency, safety, and survival outcomes in two stem cell mobilization regimens with cyclophosphamide plus G-CSF or G-CSF alone in multiple myeloma: a meta-analysis
Source: Ann Hematol. 2021 Jan 6;100(2):563–73. doi: 10.1007/s00277-020-04376-w (PMC7817584; doi:10.1007/s00277-020-04376-w)
Supplement: Supplementary file 8 — (DOCX 24 kb) [file 277_2020_4376_MOESM8_ESM.docx]

| **Supplementary Table 2. Summary Results of Sensitive Analysis.** | | | | | | | |
| --- | --- | --- | --- | --- | --- | --- | --- |
| Parameters | Participants in Analysis | Effect Sizes ^*^ | M-H Fixed-model  (Effect, 95%CI) | D-L Random-model  (Effect, 95%CI) | HKSJ Random-model  (Effect, 95%CI) | Heterogeneity | p-value **^a^** |
| OS-M | 255 | HR | **0.89 (0.54, 1.46) ^b^** | 0.89 (0.54, 1.46) | 0.88 (0.16, 5.04) | I^2^: 0%  p = 0.60 | p = 0.64 |
| OS-U | 838 | HR | **0.87 (0.67, 1.14)** | 0.87 (0.67, 1.14) | 0.90 (0.67, 1.22) | I^2^: 0%  p = 0.67 | p = 0.33 |
| OS-median | 486 | MSR | 1.04 (0.95, 1.14) | **0.99 (0.76, 1.30)** | 0.99 (0.64, 1.55) | I^2^: 87.6%  p < 0.0001 | p = 0.97 |
| 1-year OS | 709 | OR | 0.86 (0.50, 1.47) | **0.95 (0.33, 2.68)** | 0.93 (0.17, 5.11) | I^2^: 58.9%  p = 0.05 | p = 0.92 |
| 3-year OS | 709 | OR | 0.79 (0.55, 1.13) | **0.78 (0.40, 1.52)** | 0.71 (0.18, 2.79) | I^2^: 58.8%  p = 0.05 | p = 0.47 |
| 5-year OS | 621 | OR | 0.88 (0.63, 1.23) | **0.80 (0.42, 1.51)** | 0.79 (0.27, 2.35) | I^2^: 70.3%  p = 0.02 | p = 0.49 |
| PFS-M | 180 | HR | 0.57 (0.34, 0.94) | **0.57 (0.28, 1.19)** | 0.57 (0.01, 63.9) | I^2^: 51.9%  p = 0.15 | p = 0.13 |
| PFS-U | 572 | HR | 1.26 (0.98, 1.61) | **1.22 (0.80, 1.87)** | 1.61 (1.24, 2.09) | I^2^: 61.3%  p = 0.02 | p = 0.36 |
| PFS-median | 448 | MSR | 0.85 (0.78, 0.93) | **0.96 (0.53, 1.75)** | 0.96 (0.41, 2.28) | I^2^: 97.4%  p < 0.0001 | p = 0.91 |
| 1-year PFS | 572 | OR | 0.65 (0.43, 0.99) | **0.74 (0.32, 1.71)** | 0.74 (0.25, 2.20) | I^2^: 67.4%  p = 0.01 | p = 0.48 |
| 3-year PFS | 536 | OR | 0.81 (0.55, 1.19) | **1.02 (0.41, 2.56)** | 1.01 (0.30, 3.43) | I^2^: 78.2%  p = 0.001 | p = 0.96 |
| 5-year PFS | 397 | OR | 0.46 (0.28, 0.76) | **0.52 (0.21, 1.34)** | 0.53 (0.06, 4.40) | I^2^: 66.8%  p = 0.05 | p = 0.18 |
| EFS-M | 296 | HR | 0.73 (0.48, 1.09) | **0.70 (0.28, 1.74)** | 0.70 (0.002, 251.6) | I^2^: 80%  p = 0.03 | p = 0.45 |
| EFS-median | 486 | MSR | 1.20 (1.10, 1.31) | **1.15 (0.80, 1.64)** | 1.15 (0.60, 2.17) | I^2^: 93.8%  p < 0.0001 | p = 0.45 |
| 1-year EFS | 482 | OR | **1.43 (0.92, 2.23)** | 1.43 (0.92, 2.24) | 1.44 (0.84, 2.44) | I^2^: 0%  p = 0.75 | p = 0.12 |
| 5-year EFS | 482 | OR | **1.21 (0.78, 1.89)** | 1.21 (0.78, 1.89) | 1.21 (0.86, 1.72) | I^2^: 0%  p = 0.88 | p = 0.39 |
| Rate of fever during ASCT | 358 | OR | **1.64 (0.96, 2.81)** | 2.05 (0.80, 5.26) | 2.12 (0.50, 9.00) | I^2^: 40.0%  p = 0.17 | p = 0.07 |
| Rate of pneumonitis during ASCT | 211 | OR | **1.06 (0.50, 2.26)** | 1.22 (0.38, 3.90) | 1.23 (0.006, 2495.4) | I^2^: 39.5%  p = 0.20 | p = 0.87 |
| Units of RBC  infusions during ASCT | 148 | SMD | -0.43 (-0.77, -0.10) | **-0.39 (-1.12, 0.34)** | -0.39 (-5.12, 4.35) | I^2^: 78.6%  p = 0.03 | p = 0.30 |
| Days in hospital during ASCT | 148 | SMD | **-0.06 (-0.39, 0.27)** | -0.05 (-0.44, 0.34) | -0.05 (-2.58, 2.49) | I^2^: 27.9%  p = 0.24 | p = 0.72 |
| Response to CR after ASCT | 281 | OR | **0.57 (0.28, 1.15)** | 0.57 (0.28, 1.15) | 0.57 (0.04, 7.46) | I^2^: 0%  p = 0.58 | p = 0.11 |
| Response to VGPR after ASCT | 281 | OR | 0.87 (0.52, 1.44) | **0.98 (0.37, 2.63)** | 0.98 (0.002, 566.8) | I^2^: 68.5%  p = 0.07 | p = 0.98 |
| L cells recovery at day 15 after ASCT (10^9^/L) | 59 | SMD | **-0.41 (-0.95, 0.13)** | -0.41 (-0.95, 0.13) | -0.41 (-3.53, 2.70) | I^2^: 0%  p = 0.37 | p = 0.14 |
| Days of neutrophil recovery to 0.5x10^9^/L after ASCT | 491 | SMD | 0.07 (-0.11, 0.25) | **0.003 (-0.38, 0.39)** | -0.005 (-0.6, 0.62) | I^2^: 76.5%  p = 0.002 | p = 0.99 |
| Days of platelet recovery to 20x10^9^/L after ASCT | 491 | SMD | 0.007 (-0.17, 0.18) | **0.01 (-0.38, 0.41)** | 0.01 (-0.53, 0.55) | I^2^: 77.8%  p = 0.001 | p = 0.96 |
| Treat-related mortality | 353 | OR | **2.16 (0.56, 8.30)** | 2.11 (0.54, 8.21) | 2.11 (0.03, 132) | I^2^: 0%  p = 0.64 | p = 0.26 |
| ***M-H***: Mantel-Haenszel statistical method for fixed effects model. ***D-L***: DerSimonian-Laird statistical method for random effects model. ***HKSJ***: Hartung-Knapp-Sidik-Jonkman adjustment for random effects model. ***OS***: Overall survival; ***PFS***: Progression-free survival; ***EFS***: Event-free survival; ***HR***: Hazard ratio; ***OR***: Odds ratio; ***MSR***: Median survival ratio; ***M***: Multivariate; ***U***: Univariate; ***SMD***: Standard mean difference; ***ASCT***: Autologous stem cell transplantation. ***RBC***: Red blood cells. ***CR***: Complete response. ***VGPR***: Very good partial response. ***L***: Lymphocyte. **^*^** All effect sizes were calculated by comparing the CTX plus G-CSF to G-CSF alone. **^a^** The p-value of test for overall effect. **^b^** Bold values is the estimated overall effect in this study. | | | | | | | |
