# Supplementary material for: Comparison of the efficiency, safety, and survival outcomes in two stem cell mobilization regimens with cyclophosphamide plus G-CSF or G-CSF alone in multiple myeloma: a meta-analysis
Source: Ann Hematol. 2021 Jan 6;100(2):563–73. doi: 10.1007/s00277-020-04376-w (PMC7817584; doi:10.1007/s00277-020-04376-w)
Supplement: Supplementary file 9 — (DOCX 15 kb) [file 277_2020_4376_MOESM9_ESM.docx]

Pubmed = 308

((((((((multiple myeloma[MeSH Terms]) OR plasmacytoma[MeSH Terms]) OR myelomatosis[MeSH Terms]) OR leukemia, plasma cell[MeSH Terms]) OR multiple myeloma[Title/Abstract]) OR MM[Title/Abstract]) OR Kahler[Title/Abstract])) AND (((granulocyte colony-stimulating factor[MeSH Terms] OR granulocyte colony-stimulating factor[Title/Abstract] OR G-CSF[Title/Abstract])) AND (Cyclophosphamide[MeSH Terms] OR Cyclophosphamide[Title/Abstract] OR Sendoxan[Title/Abstract] OR B 518[Title/Abstract] OR Cytophosphane[Title/Abstract] OR Cytoxan[Title/Abstract] OR Endoxan[Title/Abstract] OR Neosar[Title/Abstract] OR NSC 26271[Title/Abstract] OR Procytox[Title/Abstract] OR Cyclophosphane[Title/Abstract]))

Embase = 578

('multiple myeloma':ab,ti OR plasmacytoma:ab,ti OR myelomatosis:ab,ti OR kahler:ab,ti) AND (('granulocyte colony-stimulating factor':ab,ti OR 'g csf':ab,ti) AND ' Cyclophosphamide':ab,ti)

WOS = 1135

(TS=multiple myeloma OR TS=plasmacytoma OR TS=myelomatosis OR TS=kahler) AND ((TS=Cyclophosphamide OR TS=Sendoxan OR TS=B 518 OR TS=Cytophosphane OR TS=Cytoxan OR TS=Endoxan OR TS=Neosar OR TS=NSC 26271 OR TS=Procytox OR TS=Cyclophosphane) AND (TS= granulocyte colony stimulating factor OR TS=G-CSF))

Cochrane = 141

((multiple myeloma):ti,ab,kw OR ("plasmacytoma"):ti,ab,kw OR (myelomatosis):ti,ab,kw OR ("Kahler's disease"):ti,ab,kw) AND ((("granulocyte colony stimulating factor"):ti,ab,kw OR (G-CSF):ti,ab,kw) AND ((Cyclophosphamide):ti,ab,kw OR (Sendoxan):ti,ab,kw OR (B 518):ti,ab,kw OR (Cytophosphane):ti,ab,kw OR (Cytoxan):ti,ab,kw OR (Endoxan):ti,ab,kw OR (Neosar):ti,ab,kw OR (NSC 26271):ti,ab,kw OR (Procytox):ti,ab,kw OR (Cyclophosphane):ti,ab,kw))
